# Supplementary material for: Curated and harmonised transcriptomics datasets of interstitial lung diseases
Source: Data Brief. 2025 Oct 14;63:112139. doi: 10.1016/j.dib.2025.112139 (PMC12581653; doi:10.1016/j.dib.2025.112139)
Supplement: Supplementary file 1 [file mmc1.zip › Supplementary_material/RNA-seq/fastQC reports/GSE184316/TRIMMED_SRR15926784_fastqc.html]

TRIMMED\_SRR15926784.fastq.gz FastQC Report 

FastQC Report

Sat 3 Dec 2022  
TRIMMED\_SRR15926784.fastq.gz

## Summary

- Basic Statistics
- Per base sequence quality
- Per sequence quality scores
- Per base sequence content
- Per sequence GC content
- Per base N content
- Sequence Length Distribution
- Sequence Duplication Levels
- Overrepresented sequences
- Adapter Content

## Basic Statistics

| Measure | Value |
| --- | --- |
| Filename | TRIMMED\_SRR15926784.fastq.gz |
| File type | Conventional base calls |
| Encoding | Sanger / Illumina 1.9 |
| Total Sequences | 7281316 |
| Sequences flagged as poor quality | 0 |
| Sequence length | 35-371 |
| %GC | 51 |

## Per base sequence quality

## Per sequence quality scores

## Per base sequence content

## Per sequence GC content

## Per base N content

## Sequence Length Distribution

## Sequence Duplication Levels

## Overrepresented sequences

| Sequence | Count | Percentage | Possible Source |
| --- | --- | --- | --- |
| CAGTGGGGCCACGAGCTGAGTGCGTCCTGTCACTCCACTCCCATGTCCCT | 71353 | 0.9799464822018437 | No Hit |
| GAAAGGTGTGAGTTGGCCAGAACTCTGAAAAGATTGGGAATGGATGGCTA | 50822 | 0.6979782226179992 | No Hit |
| TGATATGGTTCCTGGCAAGCCCATGTGTGTTGAGAGCTTCTCAGACTATC | 35734 | 0.490762933513667 | No Hit |
| TGATGACACCCACCGCAACTGTCTGTCTCATATCACGAACAGCAAAGCGA | 35612 | 0.4890874122205381 | No Hit |
| AGGCTACACACTGAGGACTCTGTTCCTCCCCTTTCCGCCTAGGGGAAAGT | 34536 | 0.47430986376638506 | No Hit |
| GTAACCACTCTCCCATTTGGCCAAACACATCCAGTTTGCTAGGCTGATTC | 34334 | 0.4715356399859586 | No Hit |
| CACCTCCCCGAAGCTCAGGGAGAGCCCTGTTAGGGCCGCCTCTGGCCCTA | 31201 | 0.4285077038271653 | No Hit |
| GTAACCACTCTCCCATTGGCCAAACACATCCAGTTTGCTAGGCTGATTCC | 30551 | 0.4195807461178721 | No Hit |
| GTCCAGGGTGGGCATTGTCAGGGAAGCTGCAGAGTTATTGAACCACTTGG | 29846 | 0.40989843044856183 | No Hit |
| CAAGGGTGACACCATTCAGCTCTACCTGGGGGCCAAGTTGTTGGACTCAC | 26986 | 0.3706198165276716 | No Hit |
| CACCTCCCCGAAACTCAGGGAGAGCCCTGTTAGGGCCGCCTCTGGCCCTA | 25998 | 0.35705084080954597 | No Hit |
| GGGAAGCGGGGAATGTCTACGTGCGTATGCACGTGGCACTCTCTGCCCGA | 25560 | 0.3510354446915915 | No Hit |
| CATGGCATTTGAATCATTTGAATCACTGTCATCTTCTTGTTCACTTCCTG | 21954 | 0.301511430076651 | No Hit |
| CCCTGGCTGCTACAGGAGAATAGCAGACAGGTATAGTTAAGAAACAACTT | 20049 | 0.27534857709787625 | No Hit |
| AGCTGCTAAGTGCTGTGTTGTCGTTCCCCCTGCTTAAAATAAAGTTGTTT | 19585 | 0.26897610267155003 | No Hit |
| TATCTTCTTATTTATTTTCTGCCACTGAATTCTAGATTACCAGAGTGGTT | 18903 | 0.25960966396733776 | No Hit |
| GGCAATGAGCGGTTCCGCTGCCCTGAGGCACTCTTCCAGCCTTCCTTCCT | 18698 | 0.2567942388436376 | No Hit |
| GTCCACGTCACACTTCATGATGGAGTTGAAGGTAGTTTCGTGGATGCCAC | 17056 | 0.23424337029185383 | No Hit |
| CAAGGGGGCAGACCACACAGACCTCCCCAGGGACAGCCTCCCCAGTAATC | 16974 | 0.23311720024237376 | No Hit |
| TTCAGCCCTGATCAAGACATGAGGGAGGCAGGGGCTCAGCTGAAGAAGCT | 15576 | 0.21391737427684776 | No Hit |
| CTTTGAAAGGTGTGAGTTGGCCAGAACTCTGAAAAGATTGGGAATGGATG | 15047 | 0.2066522040795922 | No Hit |
| TTACAAAAGCAGAATAAAAGCGAAAAGAAATGAAAATGTTACACTACATT | 14835 | 0.2037406424882535 | No Hit |
| ACAAGAAGTACATCAAAGATTACATGAAATCAATCAAAGGGAAACTTGAA | 14332 | 0.19683255059936966 | No Hit |
| CACCATGAAGTCCAGCGGCCTCTTCCCCTTCCTGGTGCTGCTTGCCCTGG | 14227 | 0.19539050358479154 | No Hit |
| GTCAGCCTGGAGGTCAGCCAGGGTCCTGGGCTCCTAAATGACACTAAAGT | 13395 | 0.18396399771689623 | No Hit |
| CAGCCTGCAGGAAGCATCATCATTCTTCCGGAGGGACAGACCAGCAAGAC | 12244 | 0.16815641568090164 | No Hit |
| CGAGCCACAGCTCTGCCAGTACCCCAAGACTCAGCACTAGTCTGATGACC | 12047 | 0.16545086080593124 | No Hit |
| AGCTCGTGTGTTGTAACCACTCTCCCATTGGCCAAACACATCCAGTTTGC | 11631 | 0.15973760787198357 | No Hit |
| CAGCCTGCAGGAAGCATCATCATTCTTCCAGAGGGACAGACCAGCAAGAC | 11188 | 0.153653542848573 | No Hit |
| TCTCAGTCTTGAAAGGGCAGTTGATGGTCACCGTTCTGCCCAGGTCCACT | 10949 | 0.15037116916777132 | No Hit |
| CAATCCACTTGATCCCAACTCATCTCTCATTTATTTCGGCTTCTTTTATT | 10896 | 0.14964327876993666 | No Hit |
| GTTCTGCAGCCCCTGTCATAAAAGGTTTTACTCTTTCTGGTCTCTGTTCT | 10588 | 0.14541327419384079 | No Hit |
| AGCTCGTGTGTTGTAACCACTCTCCCATTTGGCCAAACACATCCAGTTTG | 10230 | 0.1404965805631839 | No Hit |
| CAATGGGGTACTTCAGGGTCAGGATGCCACGCTTGCTCTGGGCCTCGTCG | 10227 | 0.14045537921991025 | No Hit |
| CGAAGCGGTTGTACTTGCGGATGTAGTGCAGATAGTCTCGGCGGATGACA | 9866 | 0.1354974842459797 | No Hit |
| CTCCACCTACGGCGAGGGCGAGAGCGGCCCCATGGGCAACATCATGATCG | 9700 | 0.13321767658483713 | No Hit |
| GTCTCAACATCTGTGTTGGGGAGAGTGGAGACAGACTGACGCGAGCAGCC | 9541 | 0.13103400539133309 | No Hit |
| AGAGGGCTCCCAGAAGTGTGTGGCTGAGCTGGGTCCCCAGGCCGTGGGGG | 9496 | 0.1304159852422282 | No Hit |
| CCAGTCTCGGCTCAGCCAAACACTGTCAGGGCCCCCAGCAGGGCCTTCAG | 9470 | 0.13005890693385647 | No Hit |
| TAGGAGGACAGACTCCAGCTTTGAAGGACTTTCCAGAGCCTTCCACAGCC | 9367 | 0.12864432748146076 | No Hit |
| CTCTCTTTCTGGCCTGGAGGCTATCCAGCGTACTCCAAAGATTCAGGTTT | 9273 | 0.12735335205888604 | No Hit |
| CAGCAGTTTACACCTGCTAGGGTGTTCAAAGGTCAGTGCTATAGAAATTC | 9252 | 0.12706494265597043 | No Hit |
| GGCAATGAGCGGTTCCGGTGTCCGGAGGCGCTGTTCCAGCCTTCCTTCCT | 8939 | 0.12276626917441848 | No Hit |
| GTCCACGTCACACTTCATGATGGAGTTGAAGGTGGTCTCGTGGATGCCGC | 8664 | 0.11898947937433288 | No Hit |
| CTCCGCCGCCTCCGCCGCAGACGCCGCCGCGATGCGCTACGTCGCCTCCT | 8623 | 0.11842639434959286 | No Hit |
| CAGGGTGAAGGCCCACCCGTGGAGGCCAGACCCAAAGCCCACGGTACCGA | 8602 | 0.11813798494667722 | No Hit |
| GTGTCCATTCGAGGGCGGATCCTCTCTGGCGTGGTGACCAAGATGAAGAT | 8542 | 0.11731395808120401 | No Hit |
| TTTTCCATGAGCTTAATGATGCTTTCTCTGGGCTTTTGGGGGAGGGTGTC | 8126 | 0.11160070514725634 | No Hit |
| GAAACCCAGACACATAGCAATTCAGGAAATTTGACTTTCCATTCTCTGCT | 7950 | 0.10918355967520156 | No Hit |
| CGATCAGCACGGGCTCGTACTCTGCCACAAACTGATCACACTGCTTCTGG | 7490 | 0.10286602037324023 | No Hit |
| AACAAGCACAAAGCCAAGAAAACCAACGATGCCAGATACTGAATTTCTAT | 7490 | 0.10286602037324023 | No Hit |
| CTGGAAGACGTACCTTTGTTGGAAGACGTACCTTTGTTGGAAGACGTACC | 7339 | 0.10079221942846596 | No Hit |

## Adapter Content

Produced by FastQC (version 0.11.7)
